# Toward functional precision oncology: organotypic brain slices in glioblastoma

**DOI:** 10.3389/fonc.2026.1845730

**Published:** 2026-08-21

**Authors:** Aleksandra Bondar, Susanne Raulefs, Jette Wrana, Markus Ballmann, Gerhard Rammes, Stephanie E. Combs, Thomas Ernst Schmid

**Affiliations:** 1 Department of Radiation Oncology, Technical University of Munich (TUM) School of Medicine and Health, Technical University of Munich (TUM) University Hospital, Technical University of Munich, Munich, Germany; 2 Department of Anaesthesia, Technical University of Munich (TUM) School of Medicine and Health, Technical University of Munich (TUM) University Hospital, Technical University of Munich, Munich, Germany

**Keywords:** ex vivo tissue culture, glioblastoma, organotypic brain slices, precision oncology, tumor microenvironment

## Abstract

Glioblastoma remains the most aggressive primary brain tumor in adults and is characterized by extensive invasiveness, high recurrence rates, and pronounced inter- and intratumoral heterogeneity. These features continue to limit the effectiveness of current therapies and highlight the need for experimental models that more faithfully capture the complexity of the disease. Conventional two-dimensional *in vitro* systems, while highly accessible and experimentally tractable, fail to reproduce the three-dimensional architecture, extracellular matrix organization, and dynamic tumor–microenvironment interactions that critically shape glioblastoma behavior. Although three-dimensional spheroid and organoid models partially address these limitations, they still incompletely reflect the structural and cellular complexity of native brain tissue. Organotypic brain tissue slice cultures have emerged as a powerful intermediate model that preserves native cytoarchitecture, cellular diversity, and key aspects of the tumor microenvironment. In this review, we provide an integrated overview of organotypic slice models derived from mouse, rat, and human tissue, with a focus on their application in studying glioblastoma invasion, tumor–microenvironment interactions, and therapeutic response. We discuss methodological approaches, advances in tissue preservation and bioengineering, and the integration of molecular and electrophysiological readouts. Particular emphasis is placed on human brain slice cultures, which retain patient-specific structural and cellular features and therefore offer a unique platform for investigating tumor heterogeneity and individualized treatment responses. At the same time, we address current limitations, including restricted viability, methodological variability, and the need for standardized experimental frameworks. By positioning organotypic brain slices as structurally preserved tumor ecosystems rather than simplified experimental systems, this review highlights their potential to bridge mechanistic discovery and translational application, and to contribute to the development of functionally informed precision oncology strategies in glioblastoma.

## 1 Introduction

Glioblastoma is the most aggressive primary malignant brain tumor in adults and continues to carry a poor prognosis despite intensive multimodal treatment approaches (1–4). Recurrence occurs in the vast majority of patients, typically close to the original tumor site, reflecting the highly infiltrative nature of the disease. This diffuse growth pattern, together with pronounced inter- and intratumoral heterogeneity, underlies therapeutic resistance and highlights the need for experimental models that better capture the biological complexity of glioblastoma.

Conventional two-dimensional cell culture systems have been instrumental for studying proliferation, signaling pathways, genetic manipulation, and drug responses. However, they fail to recapitulate key features of the *in vivo* tumor environment, including three-dimensional architecture, cell polarity, extracellular matrix organization, and long-range cellular interactions. Three-dimensional systems such as spheroids and organoids offer some improvement, yet they still lack essential components of the brain microenvironment, including vascular perfusion, physiologically relevant oxygen and nutrient gradients, and the full spectrum of neural and immune cell interactions (5).

Ex vivo brain tissue slice cultures provide an alternative approach that retains much of the native tissue organization. Typically prepared at thicknesses of around 300–400 µm, these slices preserve structural integrity while allowing sufficient diffusion of oxygen and nutrients (6, 7). Multiple slices can be obtained from a single donor, making this system compatible with the principles of Replacement, Reduction, and Refinement. Importantly, organotypic slices maintain the spatial organization of neurons, glial cells, vasculature, and extracellular matrix, enabling tumor cells to interact with a more physiologically relevant environment. Glioblastoma cells can be introduced into slices derived from healthy rodent brain tissue or studied in tumor-bearing slices generated *in vivo* (8–10). In addition, human brain slices obtained from surgical resections allow investigation of tumor behavior in a patient-specific context, including invasion patterns and treatment responses (11).

Methodological approaches used in organotypic brain slice cultures vary substantially across studies and critically influence tissue preservation, tumor behavior, and experimental reproducibility. In most protocols, brain tissue is sectioned into slices approximately 250–400 µm thick using a vibratome in ice-cold oxygenated artificial cerebrospinal fluid or protective choline-based solutions to minimize mechanical and metabolic stress. Slices are commonly maintained either at the air–liquid interface on semipermeable membrane inserts or in submerged culture systems, with the air–liquid interface generally providing improved oxygen and nutrient diffusion for long-term viability. Glioblastoma cells are introduced using different approaches, including implantation of fluorescently labeled spheroids, direct microinjection of dissociated cells, or the use of tumor-bearing tissue derived *in vivo*. Viability is assessed using a combination of histological preservation, live/dead staining, lactate dehydrogenase release assays, electrophysiological recordings, and molecular profiling approaches. In addition, advanced imaging techniques such as confocal microscopy, multiphoton microscopy, and live-cell time-lapse imaging are increasingly combined with transcriptomic and spatial analyses to investigate invasion dynamics and tumor–microenvironment interactions in preserved tissue contexts. Despite these advances, considerable methodological heterogeneity remains regarding oxygenation conditions, media composition, culture duration, and endpoint quantification, highlighting the need for standardized experimental and reporting frameworks to improve reproducibility and translational comparability across studies.

Despite these advantages, slice cultures are not without limitations. Tissue viability is restricted, with murine slices typically maintained for about one week and human slices for longer periods under optimized conditions (12–14). Over time, changes in tissue physiology and progressive cell loss can influence experimental outcomes and must be carefully considered (15). Diffusion constraints and variability in the preservation of stromal and immune components further complicate interpretation. The intrinsic heterogeneity of glioblastoma adds another layer of complexity. Differences between patients, as well as spatial variability within individual tumors, can lead to divergent biological behavior and treatment responses (16, 17). While this variability poses challenges, it also provides an opportunity to investigate regional tumor evolution and mechanisms of resistance within a controlled experimental setting.

A recent systematic review in Cancers offered a structured, PRISMA-based overview of ex vivo brain slice models, focusing primarily on methodological classification and study heterogeneity (18). Building on this foundation, the present review takes a more integrative approach. We consider organotypic brain slices not simply as experimental tools, but as structurally preserved tumor ecosystems. By linking tissue architecture to invasion dynamics, microenvironmental signaling, immune interactions, and therapeutic response, we aim to provide a more mechanistic perspective on their role in glioblastoma research.

**Figure 1:**
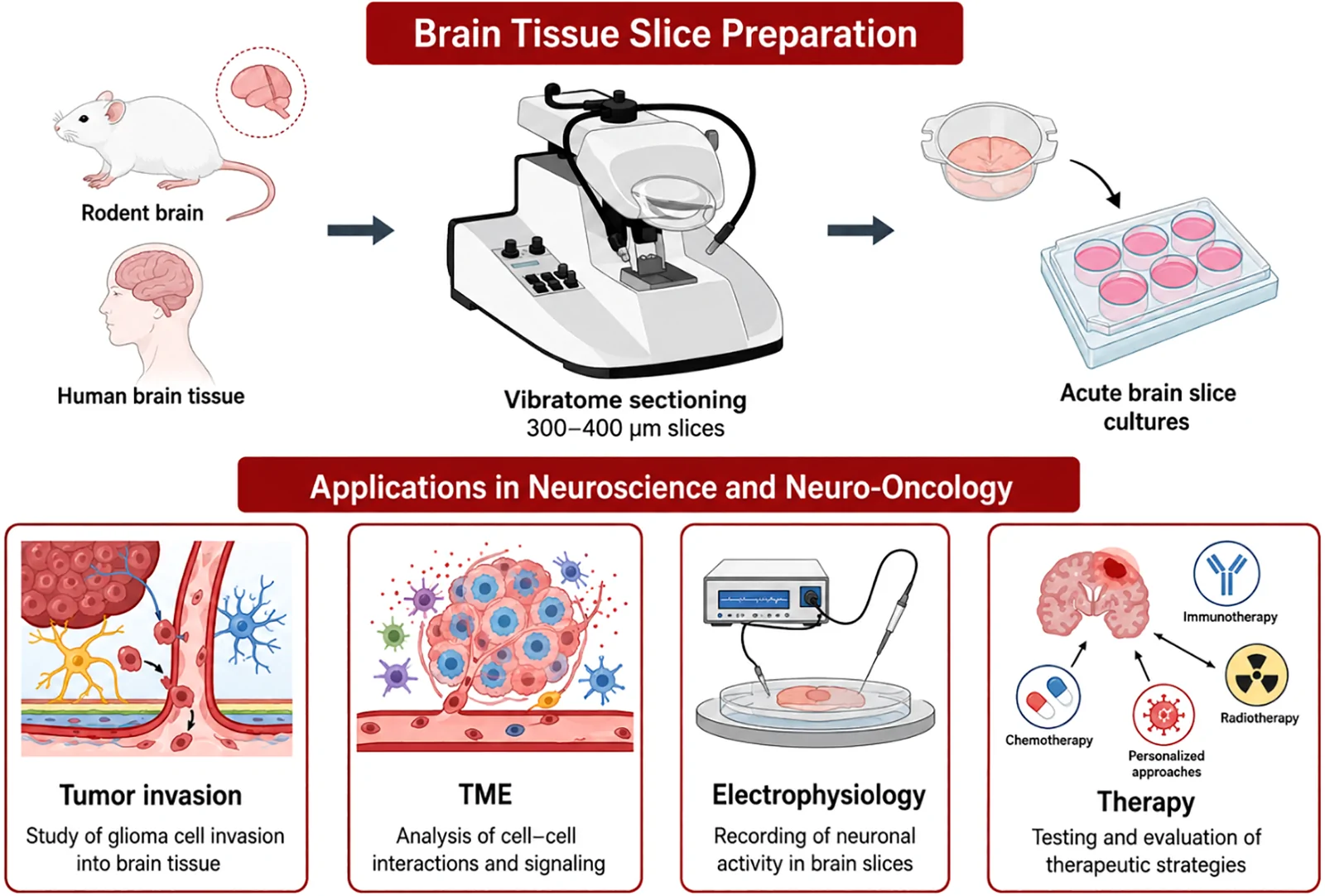
Schematic representation of organotypic brain slice preparation and applications. Created with BioRender.com (https://BioRender.com/y1b6qcw).

In this context, we synthesize findings from mouse, rat, and human brain slice models within a unified framework (**Figure 1**), emphasizing their complementary strengths and their potential to bridge experimental neuro-oncology and translational application.

An overview of representative studies using organotypic brain slice cultures in glioblastoma research is summarized in **Table 1**, highlighting the experimental models, methodological approaches, and major applications across rodent and human systems.

**Table 1:** Overview of representative studies using organotypic brain slice cultures in glioblastoma research, grouped according to species/model system and including the principal experimental strengths of each approach.

Study	Species/model	Experimental approach	Main application/findings	Key purpose/model strength
Mouse-derived slice models				
Tobias Ohnishi et al. (8)	Rat brain slices	Implantation of glioma cells into organotypic brain slices	Early invasion model demonstrating glioma infiltration in preserved brain tissue	Foundational proof-of-concept invasion model
Thomas Eisemann et al. (19)	Adult mouse slices (C57BL/6)	Fluorescent glioblastoma spheroids implanted into slices	Quantification and visualization of glioblastoma invasion dynamics	High-resolution invasion quantification
Shawn Watkins & Harald Sontheimer (20)	Mouse slices (C.B-17 scid)	EGFP-labeled glioblastoma cells with live imaging	Demonstrated hydrodynamic volume changes during invasion	Live-cell imaging of invasion mechanisms
Shawn Watkins et al. (21)	Mouse slices	Glioblastoma implantation with astrocyte and vascular imaging	Showed disruption of astrocyte–vascular coupling and BBB integrity	Investigation of perivascular invasion
Pavel Gritsenko et al. (22)	Mouse slices + biomimetic matrices	Tissue-inspired invasion platforms	Analysis of glioma migration along vessels and astrocyte-rich regions	Modeling ECM-guided migration
Laurent Decotret et al. (23)	Mouse slices (C57BL/6J)	Agar-embedded slice invasion assay	Improved deep-tissue imaging and quantification of invasion depth	Enhanced imaging and spatial analysis
Yuan Huang et al. (24)	Humanized mouse slices	Human iPSC-derived microglia and glioma cells transplanted into mouse slices	Investigation of immune–tumor interactions via CCL18/CCR8 signaling	Human immune–tumor interaction model
Maria Angeles et al. (25)	Mouse slices	Region-specific implantation of glioblastoma stem cells	Demonstrated microenvironment-dependent stemness and differentiation	Anatomical niche-specific tumor behavior
Wei-Li Xu et al. (26)	Postnatal mouse slices	U251MG spheroid implantation with NKCC1 inhibition	Drug testing of invasion-associated ion transport pathways	Pharmacological invasion studies
Naoki Minami et al. (27)	Mouse tumor-bearing slices	Ex vivo explant cultures treated with chemotherapeutics	Evaluation of therapy-induced morphological and viability changes	Therapy response assessment in preserved tissue
Verena Nickl et al. (28)	Mouse hippocampal slices	Patient-derived glioblastoma cells exposed to TTFields	Assessment of anti-proliferative effects of Tumor Treating Fields	Functional therapy testing platform
Thomas Suckert et al. (29)	Tumor slice cultures	Proton irradiation studies	Analysis of radiation-induced DNA damage and microenvironment preservation	Radiobiological modeling
Rat-derived slice models				
Christine Beadle et al. (30)	Rat slices	Glioblastoma invasion assays	Identified myosin II–dependent invasion in confined tissue spaces	Mechanobiology of invasion
Camilla Aaberg-Jessen et al. (31)	Rat corticostriatal slices	Implantation of patient-derived spheroids	Comparison of invasive behavior between primary and U87MG spheroids	Translational comparison of tumor models
Sara Petterson et al. (32)	Rat slices	Irradiated glioblastoma spheroid implantation	Radiobiological evaluation of invasion and proliferation	Ex vivo irradiation response model
Amin Ghoochani et al. (33)	Rat slices	Vascular Organotypic Glioma Impact Model	Quantification of angiogenesis, microglial recruitment, and TMZ response	Tumor–vascular interaction analysis
Jan Hörnschemeyer et al. (34)	Rat slices	Kinase inhibitor testing in implanted glioblastoma models	Combined assessment of tumor viability and neuronal activity	Integrated therapy and electrophysiology platform
Human-derived slice models				
Seung U. Jung et al. (35)	Human brain slices	Implantation of fluorescent glioblastoma cells/spheroids	Dynamic tracking of glioblastoma migration in human tissue	Human-specific invasion analysis
John J. Parker et al. (11)	Human glioblastoma slices	ZsGreen-labeled tumor cells in patient-derived slices	Patient-specific invasion analysis and gefitinib response	Personalized invasion and drug-response profiling
Vijay Ravi et al. (36)	Human cortical slices	Patient-derived glioblastoma implantation with MEA recordings	Investigation of tumor growth, invasion, and electrophysiological activity	Combined functional and electrophysiological analysis
Anders Bak et al. (37)	Human slices	Optimized long-term slice culture protocol	Extended viability and preservation of neuronal function	Long-term tissue preservation
Bas Plug et al. (38)	Human post-mortem slices	Long-term organotypic culture system	Viability maintenance for up to six weeks	Extended ex vivo human tissue platform
Dirk Henrik Heiland et al. (39)	Human glioblastoma slices	Analysis of microglia–astrocyte interactions	Identification of immunosuppressive microenvironment signaling	Tumor–immune microenvironment modeling
Florian Merz et al. (40)	Human glioblastoma slices	Chemoradiation testing ex vivo	Patient-specific therapeutic susceptibility analysis	Functional precision oncology platform
Ugne Kuliesiute et al. (41)	Human slices	Metabolic and transcriptional analyses	Investigation of sialic acid metabolism and tumor connectivity	Mechanistic metabolic profiling
Wei Zhao et al. (42)	Acute human tumor slices	Single-cell RNA-seq with multiplex drug perturbation	Cell type-specific drug response profiling in preserved tissue	High-throughput functional precision medicine

## 2 Mouse brain tissue slices

Mouse brain slice cultures are widely used in neuro-oncology because they combine excellent experimental accessibility with a rich repertoire of genetic models. Compared with two-dimensional cell culture, mouse slices preserve the native three-dimensional tissue architecture and the cellular network that shapes glioblastoma invasion, proliferation, and microenvironmental remodeling. This makes mouse slices valuable for studying glioblastoma behavior under conditions that maintain neuronal and glial interactions.

### 2.1 Invasion visualization models

Organotypic mouse brain slices serve as powerful platforms for studies of glioblastoma invasion. Eisemann and colleagues developed an assay in which fluorescently labeled glioblastoma spheroids from human and mouse cell lines were implanted into slices prepared from adult C57BL/6 mice (19). Lipophilic fluorescent dyes enabled precise visualization of tumor infiltration using fluorescence microscopy. This model has been used to examine molecular drivers of invasion and to quantify how genetic perturbations or pharmacological agents affect cell motility.

Dankner and co-workers used organotypic slices from tdTomato-expressing mice to explore interactions between infiltrating metastatic cancer cells and reactive astrocytes (43). Although the mouse strain was not specified in the publication, the fluorescent labeling allowed direct visualization of host–tumor interactions. Astrocytic STAT3 activation promoted invasive outgrowth through secretion of CHI3L1, and astrocyte-specific deletion of STAT3 reduced invasive behavior. These results highlight how organotypic slices can reveal cross-talk between glioblastoma and the surrounding neural tissue.

### 2.2 Invasion pathway models

Watkins and Sontheimer used immunodeficient C.B-17 scid mouse slices to study how hydrodynamic volume changes enable glioblastoma cells to navigate tight extracellular spaces (20). Glioblastoma cells expressing enhanced green fluorescent protein were observed by time-lapse confocal and multiphoton microscopy. The authors demonstrated that invading cells undergo marked volume fluctuations as they move through narrow extracellular corridors. This was regulated by ion channel activity and osmotic gradients.

In a complementary study, Watkins and colleagues evaluated how perivascular invasion alters astrocyte and vascular interactions using Gl261 glioma cells, patient-derived xenograft lines, and non-malignant Aldh1l1-EGFP glia (21). Perivascular glioblastoma cells displaced astrocytic endfeet and disrupted blood–brain barrier properties. These models illustrate how organotypic slices can connect microscopic invasion dynamics to functional changes in brain physiology.

Gritsenko and co-workers integrated organotypic slices with tissue-inspired matrices to model invasion along blood vessels and within astrocyte-rich regions. Their results demonstrated that glioblastoma cells migrate cohesively at speeds consistent with *in vivo* observations and that matrix composition profoundly influences migration patterns (22).

Decotret and colleagues improved deep-tissue imaging by embedding brain slices in agar prior to sectioning. Slices prepared from six-week-old C57BL/6J mice supported rapid infiltration of implanted glioblastoma spheroids and enabled quantitative assessment of invasion depth and vascular interactions (23).

Huang and colleagues created a hybrid humanized slice model using murine brain slices depleted of endogenous microglia, into which human induced pluripotent stem cell-derived microglia and human glioblastoma cells were transplanted to study immune signaling within the tumor microenvironment (24). In this system, microglia secreting CCL18 promoted glioblastoma proliferation and invasion through an ACP5-mediated signaling cascade. This approach provides a valuable platform for investigating human immune–tumor interactions in a controlled ex vivo environment. However, potential limitations and artifacts associated with cross-species systems must be considered carefully. Species-specific differences in extracellular matrix composition, cytokine signaling, immune responses, and neuronal–glial interactions may influence tumor behavior and microenvironmental dynamics. Therefore, although such hybrid models offer important mechanistic insights, their translational interpretation requires cautious evaluation.

### 2.3 Tumor microenvironment models

Angeles and colleagues used mouse brain slices from young adult animals to compare how glioblastoma stem cells behave when implanted into different anatomical regions including the subependymal zone, corpus callosum, striatum, and cortex (25). Cells placed into the subependymal zone showed enhanced proliferation and expression of stem cell markers, whereas those implanted into cortical or striatal tissue underwent partial differentiation. These findings demonstrate how region-specific cues in the brain shape glioblastoma cell behavior and can be analyzed effectively in slice cultures.

Completing these observations, Yalcin et al. showed that tumor growth itself is critically dependent on signals that come from the tumor microenvironment. In their ex vivo brain slice experiments, the selective loss of the microglia/macrophage-derived factor GPNMB resulted in a significant reduction in tumor growth, independent of tumor-intrinsic properties such as the intrinsic GPNMB expression of glioma cells (44).

### 2.4 Therapy testing in mouse slices

Organotypic slices enable systematic testing of candidate therapeutics under physiologically relevant conditions. Xu and colleagues used postnatal mouse slices implanted with U251MG spheroids to evaluate inhibitors of chloride and potassium cotransport. The NKCC1 inhibitor bumetanide reduced invasion and decreased phospho-NKCC1 expression, demonstrating the suitability of this model for preclinical drug assessment (26).

Minami and co-workers developed tumor-bearing slices by introducing oncogenic neural progenitor cells into adult C57BL/6J mice prior to slice preparation (27). These explants preserved tumor–host interactions and were treated with agents including cisplatin, temozolomide, paclitaxel, and tranilast. The system enabled direct comparison of cytotoxic and cytostatic effects and provided valuable insights into therapy-induced changes in tumor morphology and viability.

Nickl and colleagues used organotypic hippocampal slices to study glioblastoma responses to Tumor Treating Fields. Slices implanted with patient-derived glioblastoma cells responded to alternating electric fields with decreased proliferation, although responses varied between patients (28).

Suckert and co-workers compared tumor slice cultures with adult organotypic slices to study proton beam irradiation. Tumor slice cultures maintained viability and microenvironmental architecture more effectively and displayed radiation-induced DNA damage patterns consistent with *in vivo* responses (29).

### 2.5 Electrophysiology models

Electrophysiological recordings in organotypic slices provide valuable information on how glioblastoma alters neuronal network activity. Methods developed for acute slice physiology, including the N-methyl-D-glucamine protective recovery protocol and microfluidic slice-on-a-chip platforms, improve tissue preservation and enable long-term monitoring of neural activity (45, 46). These approaches can be applied to glioblastoma-bearing slices to study tumor-induced hyperexcitability, alterations in synaptic connectivity, and responses to therapeutic interventions.

## 3 Rodent brain tissue slices

Rodent-derived organotypic brain slice cultures are widely used in neuro-oncology because they combine experimental accessibility with preservation of the native three-dimensional tissue architecture and cellular microenvironment. Compared with conventional two-dimensional culture systems, rodent slices retain neuronal and glial networks, extracellular matrix structures, and vascular components that critically influence glioblastoma invasion, proliferation, and therapy response. Mouse and rat slice systems each offer specific methodological advantages. Mouse models provide extensive genetic tractability through the availability of transgenic and knockout strains, whereas rat slices are structurally larger and mechanically more robust, facilitating electrophysiological recordings, microinjection approaches, and deep-tissue imaging. Together, rodent-derived organotypic slice models represent versatile experimental platforms for studying glioblastoma biology under physiologically relevant ex vivo conditions.

### 3.1 Invasion and migration models

Organotypic rodent brain slices serve as powerful platforms for studies of glioblastoma invasion and migration. Eisemann and colleagues developed an assay in which fluorescently labeled glioblastoma spheroids from human and mouse cell lines were implanted into slices prepared from adult C57BL/6 mice (19). Lipophilic fluorescent dyes enabled precise visualization of tumor infiltration using fluorescence microscopy. This model has been used to examine molecular drivers of invasion and to quantify how genetic perturbations or pharmacological agents affect cell motility.

Similarly, Beadle and colleagues investigated glioblastoma invasion using organotypic rat brain slices combined with *in vivo* and *in vitro* assays (30). Their work demonstrated that glioblastoma invasion depends strongly on the physical environment. In confined three-dimensional spaces or dense brain tissue, glioblastoma cells relied on myosin II-mediated contractility to migrate through narrow extracellular spaces, highlighting the importance of physiologically relevant tissue architecture.

Aaberg-Jessen and co-workers established a corticostriatal rat slice model using primary glioblastoma spheroids and demonstrated that patient-derived spheroids preserved stem cell marker expression and invasive behavior more effectively than conventional U87MG spheroids (31). These findings emphasized the translational relevance of patient-derived material in organotypic systems.

Watkins and Sontheimer used immunodeficient C.B-17 scid mouse slices to study how hydrodynamic volume changes facilitate glioblastoma invasion (20). Using time-lapse confocal and multiphoton microscopy, they demonstrated that glioblastoma cells dynamically regulate cellular volume through ion channel activity to navigate narrow extracellular corridors.

Complementing these findings, Watkins and colleagues later showed that invading glioblastoma cells disrupt astrocyte–vascular interactions and blood–brain barrier integrity during perivascular invasion (21). Gritsenko and co-workers further demonstrated that extracellular matrix composition critically influences migration patterns along vascular and astrocyte-rich structures (22).

Decotret and colleagues improved deep-tissue imaging by embedding mouse brain slices in agar prior to sectioning, enabling quantitative assessment of glioblastoma invasion depth and vascular interactions (23).

### 3.2 Tumor microenvironment models

Rodent-derived organotypic slice cultures provide important opportunities to study glioblastoma–microenvironment interactions in preserved neural tissue. Angeles and colleagues demonstrated that glioblastoma stem cell behavior differs depending on the anatomical implantation site within mouse brain slices (25). Cells implanted into the subependymal zone displayed enhanced proliferation and stemness, whereas implantation into cortical or striatal regions promoted partial differentiation.

Yalcin and co-workers further showed that tumor-associated microglia/macrophages critically regulate glioblastoma growth through secretion of GPNMB, independent of tumor-intrinsic expression patterns (44).

Huang and colleagues created a hybrid humanized rodent-derived organotypic slice model using murine brain slices depleted of endogenous microglia, into which human induced pluripotent stem cell-derived microglia and human glioblastoma cells were transplanted to study immune signaling within the tumor microenvironment (24). In this system, microglia secreting CCL18 promoted glioblastoma proliferation and invasion through an ACP5-mediated signaling cascade. This approach provides a valuable platform for investigating human immune–tumor interactions in a controlled ex vivo environment. However, potential limitations and artifacts associated with cross-species systems must be considered carefully. Species-specific differences in extracellular matrix composition, cytokine signaling, immune responses, and neuronal–glial interactions may influence tumor behavior and microenvironmental dynamics. Therefore, although such hybrid models offer important mechanistic insights, their translational interpretation requires cautious evaluation.

Ghoochani and colleagues developed the Vascular Organotypic Glioma Impact Model using rat brain slices implanted with glioblastoma cells (33). Tumor implantation induced angiogenesis, microglial recruitment, and peritumoral cell death, while temozolomide treatment partially normalized these pathological alterations.

### 3.3 Therapy and radiobiological models

Rodent-derived organotypic slice cultures also serve as valuable platforms for therapeutic testing and radiobiological investigations. Xu and colleagues used postnatal mouse slices implanted with U251MG spheroids to evaluate inhibitors targeting chloride and potassium cotransport pathways (26). The NKCC1 inhibitor bumetanide significantly reduced glioblastoma invasion.

Minami and co-workers generated tumor-bearing explant cultures from adult C57BL/6J mice and evaluated responses to chemotherapeutic agents including cisplatin, temozolomide, paclitaxel, and tranilast (27). The model enabled direct comparison of cytotoxic and cytostatic effects within preserved tissue architecture.

Petterson and colleagues investigated radiation effects in rat brain slice cultures implanted with glioblastoma spheroids (32). Mature slice cultures proved more stable and suitable for radiobiological analyses than immature slices. Although irradiation reduced tumor proliferation, invasive behavior persisted, reflecting clinically relevant treatment resistance mechanisms.

Suckert and colleagues applied tissue slice cultures to proton irradiation studies and demonstrated radiation-induced DNA damage patterns consistent with *in vivo* observations (29).

Nickl and co-workers used organotypic hippocampal slices implanted with patient-derived glioblastoma cells to evaluate Tumor Treating Fields (TTFields) (28). Alternating electric fields reduced proliferation in a patient-dependent manner.

Hörnschemeyer and colleagues combined kinase inhibitor testing with rat slice implantation models, enabling simultaneous assessment of tumor viability and neuronal network activity (34).

### 3.4 Electrophysiological and technical advances

Electrophysiological recordings in rodent-derived organotypic slices provide important insights into how glioblastoma alters neuronal activity and network function. Techniques such as multi-electrode array recordings, microfluidic slice-on-a-chip systems, and optimized N-methyl-D-glucamine protective recovery protocols improve tissue preservation and enable long-term functional analyses (45, 46). These approaches facilitate investigation of tumor-induced hyperexcitability, synaptic dysfunction, and electrophysiological responses to therapeutic interventions.

### 3.5 Conceptual limitations of rodent-derived slice models

Despite their advantages, rodent-derived organotypic slice cultures remain limited by species-specific differences in brain architecture, immune composition, extracellular matrix organization, and metabolic regulation. Neonatal rodent slices are commonly used because of their improved viability and technical robustness, yet they differ substantially from adult human brain tissue in terms of cellular composition, synaptic organization, and inflammatory signaling. Adult rodent slices better reflect mature tissue conditions but are technically more challenging and more susceptible to degeneration during prolonged culture (47).

Furthermore, rodent tissue cannot fully reproduce the complexity and heterogeneity of the human glioblastoma microenvironment. Differences in white matter organization, vascular structure, immune signaling, and extracellular matrix composition may significantly influence tumor invasion and therapeutic response. Consequently, rodent-derived slice systems are particularly valuable for mechanistic and hypothesis-driven experimentation, whereas human slice cultures provide greater translational relevance and patient-specific biological fidelity. A comparative overview of the translational strengths and limitations of mouse, rat, and human organotypic slice systems is summarized in **Table 2**.

**Table 2:** Translational roles of organotypic brain slice models in glioblastoma research.

Dimension	Mouse slices	Rat slices	Human slices
Genetic tractability	Extensive transgenic and knockout models available	More limited compared to mouse	Primarily ex vivo manipulation (e.g., viral transduction)
Microenvironment fidelity	Preserved rodent brain architecture	Structurally robust, but species-specific	Native human cytoarchitecture and microenvironmental niches
Immune context	Rodent microglia and immune signaling	Rodent immune environment	Patient-specific immune milieu
Typical tissue maturity	Commonly neonatal; adult tissue less stable	Frequently neonatal	Adult clinical tissue
Viability window	~7–10 days	~7–14 days	~10–21 days (under optimized conditions)
Invasion modeling	High mechanistic resolution and control	Larger tissue volume enables broader implantation	Recapitulates human-specific invasion patterns (e.g., white matter tracts)
Primary role in research	Mechanistic discovery platform	Intermediate validation platform	Functional precision and translational platform

## 4 Human brain tissue slices

Human brain tissue slices provide the most clinically relevant platform for studying glioblastoma because they preserve patient-specific microenvironmental factors that are not replicated in rodent tissue. These slices can be derived from tumor resections, peritumoral regions, or non-tumorous neurosurgical specimens obtained during procedures for epilepsy or trauma.

A recent review by Joseph and colleagues discussed ethical, technical, and translational considerations related to the use of human brain tissue cultures, including informed consent, tissue handling, and extended use of living human samples (48). Human slices offer unparalleled opportunities for personalized medicine, although their preparation is limited by availability of surgical tissue, variability among specimens, and the need for stringent handling protocols that preserve viability.

### 4.1 Viability improvement protocols

Parker and co-workers generated human organotypic glioblastoma slices from more than fifty patients and retrovirally labeled tumor cells with ZsGreen to track migration (11). The slices preserved pathological hallmarks and microglia for at least ten to fifteen days. Hypoxia induced vascular endothelial growth factor secretion, confirming functional reactivity. The authors observed substantial patient-specific variability in invasion patterns and demonstrated that migration was suppressed by the epidermal growth factor receptor inhibitor gefitinib.

Ravi and colleagues developed a protocol that maintained human cortical slices for up to fourteen days with preserved extracellular electrical activity measured using multi-electrode arrays (36). Implantation of patient-derived glioblastoma cells reproduced clinically relevant phenotypes. Mesenchymal glioblastoma stem cells displayed diffuse infiltration, while proneural lines exhibited more circumscribed growth and greater sensitivity to temozolomide. Microglia, astrocytes, vasculature, and cytokine responses remained intact.

Bak and co-workers extended slice viability to approximately three weeks by optimizing all stages of preparation, including resection, transport, slicing in choline-based solution, and culture at the air–liquid interface in human cerebrospinal fluid (37). Viability was confirmed through viral gene expression, patch-clamp recordings, and multi-electrode array measurements. These protocols support both long-term glioblastoma studies and applications in epilepsy or neurodegeneration.

Plug and colleagues introduced human post-mortem organotypic slice cultures that remained viable for at least six weeks while maintaining structural and cellular integrity (38). Supplementation with human cerebrospinal fluid improved recovery. These slices preserved neuropathology and responded to demyelinating lesions, indicating potential uses beyond oncology. Their compatibility with viral transduction and electrophysiological methods suggests that they could support extended glioblastoma research when fresh surgical tissue is limited.

### 4.2 Invasion and migration models

Human slices provide a platform for studying invasion within authentic human cytoarchitecture. Jung and colleagues developed one of the earliest human slice models by preparing slices from non-tumorous surgical tissue and implanting either labeled patient-derived glioblastoma spheroids or fluorescent astrocytoma cells (35). The slices supported dynamic analyses of single-cell migration trajectories and showed heterogeneous, multidirectional invasion patterns that closely resembled *in vivo* tumor behavior.

### 4.3 Tumor microenvironment models

Human organotypic brain slice cultures provide an ex vivo system to study the glioblastoma tumor microenvironment in native human tissue. In the study mentioned above, Ravi et al. not only focused on enhancing the viability of brain tissue slices in culture but also revealed a direct insight into how the glioblastoma tumor microenvironment shapes tumor behavior in human tissue (36). In their setting, glioblastoma cells did not invade randomly. They followed pre-existing white-matter tracts and collagen IV–positive perivascular structures. That highlights the instructive role of native extracellular matrix organization and vascular architecture. The tumor implantation further triggered changes in the local cytokine milieu, including increased secretion of G-CSF and TGF-β, reflecting an active tumor–microenvironment signaling. Importantly, temozolomide response in this model depended on the MGMT (O-6-Methylguanin-DNA-Methyltransferase) status of the tumor cells, showing that treatment sensitivity arises from the interaction between tumor-intrinsic features and the preserved human microenvironment.

Using this slice-based model, Heiland et al. showed that microglia are key regulators of the glioblastoma tumor microenvironment (39). Microglia were required to induce a tumor-associated reactive astrocyte state, which is marked by JAK/STAT activation and an increased expression of immune and complement genes. This glial interaction induced the secretion of immunosuppressive cytokines, particularly IL-10. The disruption of microglia or the inhibition of JAK/STAT signaling reduced the tumor spread. That reveals a microglia–astrocyte axis that drives the formation of an immunosuppressive tumor microenvironment in human glioblastoma.

Nickl and co-workers compared patient-derived organoids with organotypic slices. Although organoids maintained some tumor properties, they did not retain immune or stromal components beyond early culture stages, whereas slices preserved tumor microenvironment architecture for up to nine days (49). To extend the lifespan of microenvironmental features in organoids, the authors developed enriched organoids that included peripheral blood mononuclear cells. This approach enabled preservation of immune markers for up to three weeks and supported studies of immunotherapeutic responses.

### 4.4 Personalized medicine models

Merz and colleagues used organotypic slices of human glioblastoma to evaluate individual responses to chemoradiation. Treatment with temozolomide, X-rays, or carbon ions reduced proliferation and increased DNA damage, measured by histology and γH2AX immunostaining (40). The slices preserved native histopathology for more than sixteen days and enabled quantification of treatment-specific effects. This model has significant potential for personalized therapy testing, especially when combined with molecular profiling.

### 4.5 Molecular mechanisms in human slices

Kuliesiute and co-workers applied metabolic labeling and transcriptional analysis to investigate sialic acid metabolism in glioblastoma cells within human slices (41). Glioblastoma cells exhibited high rates of sialic acid synthesis and turnover. Enzymes involved in sialylation and desialylation were highly expressed, and perturbing these pathways altered tumor network connectivity and invasive behavior. These findings highlight the use of human slices for mechanistic studies that require intact microenvironmental contexts.

Zhang and colleagues developed a workflow for isolating viable glioblastoma and microenvironmental cell populations from human slices for single-cell transcriptomics (50). Their method yielded tens of thousands of high-quality cells and enabled reconstruction of tumor–microenvironment interactions based on gene expression.

Zhao and co-workers (51) combined acute tumor slices with single-cell sequencing and multiplexed drug perturbation to evaluate cell type specific treatment responses (42). Acute human slices retained the heterogeneity of the original tumor, and this technique allowed rapid evaluation of both conserved and patient-specific drug sensitivities.

## 5 Discussion

Organotypic brain slice cultures have emerged as powerful experimental systems for investigating glioblastoma invasion, tumor–microenvironment interactions, and therapy response under conditions that preserve essential aspects of native brain architecture. By maintaining cytoarchitecture, white matter tracts, perivascular niches, and glial networks, these models provide biological insights that are not accessible in reductionist two-dimensional systems or simplified three-dimensional cultures. Particularly in human-derived slices, the preservation of patient-specific microenvironmental features enables functional interrogation of tumor heterogeneity and individualized therapy response.

At the same time, organotypic systems remain experimentally accessible and compatible with advanced molecular, imaging, and electrophysiological techniques. The integration of single-cell transcriptomics, spatial transcriptomics, metabolic labeling, and live imaging within preserved tissue contexts allows dynamic investigation of tumor cell states and niche-dependent behaviors. These capabilities position slice cultures as uniquely suited platforms at the interface between mechanistic discovery and translational application.

Despite these strengths, the field remains constrained by substantial methodological heterogeneity that limits comparability and reproducibility across studies. Variability in slice thickness, oxygenation control, culture configuration, and viability assessment does not merely represent technical nuance; it fundamentally shapes biological interpretation. Differences in slice thickness directly influence oxygen and nutrient diffusion, alter the development of hypoxic gradients, and determine the degree of cytoarchitectural preservation. These parameters, in turn, modulate invasion dynamics, proliferation rates, immune cell retention, and therapeutic response. Consequently, findings obtained across laboratories are often difficult to compare, even when similar tumor models are employed.

Oxygenation represents a particularly underrecognized variable. In many studies, oxygen levels are insufficiently controlled or inadequately reported. Given the central role of hypoxia in regulating glioblastoma stemness, invasion, metabolic adaptation, and treatment resistance, uncontrolled oxygen tension may introduce artificial metabolic stress that confounds mechanistic conclusions. Without standardized reporting of oxygen conditions, it remains challenging to distinguish biologically meaningful hypoxia-driven phenomena from culture-induced artifacts.

Viability assessment further illustrates the need for harmonization. Current approaches range from morphological inspection and histological preservation to live/dead staining, LDH release assays, apoptotic markers, electrophysiological recordings, or transcriptomic integrity analyses. The absence of clearly defined and consistently applied viability criteria undermines reproducibility and complicates translational interpretation. If organotypic slice cultures are to mature from exploratory experimental tools into robust translational platforms, methodological rigor and reporting transparency must become field-wide priorities rather than optional refinements.

Beyond methodological considerations, intrinsic limitations of slice cultures must also be acknowledged. Although organotypic systems preserve local architecture, they lack systemic circulation, dynamic immune trafficking, and long-term vascular perfusion. Over time, changes in cellular composition, metabolic state, and inflammatory signaling may occur, particularly in prolonged cultures. These factors necessitate careful interpretation of temporal effects and reinforce the importance of clearly defined experimental windows.

To address these challenges, the establishment of minimal reporting standards should be considered. Key parameters—including slice thickness range, time from resection to culture, oxygen tension, media composition, culture configuration (submerged versus air–liquid interface), and validated duration of viability—should be systematically documented. Standardized viability validation combining morphological assessment with at least one quantitative readout, such as live/dead staining, LDH release measurement, or electrophysiological activity, would enhance cross-study comparability.

Moreover, consensus recommendations for optimal slice thickness within diffusion-compatible ranges, controlled oxygen conditions approximating physiological brain levels, and clearly defined endpoints for invasion quantification could significantly improve inter-laboratory reproducibility. The development of shared protocols, open methodological repositories, and multi-center benchmarking studies may further accelerate the transition of organotypic slice models toward reproducible and clinically credible research infrastructures. Importantly, these methodological refinements would not merely improve technical consistency but would strengthen the translational relevance of organotypic systems. As slice-based platforms become increasingly integrated with molecular diagnostics, spatial multi-omics, and functional perturbation assays, standardization will be essential to ensure that ex vivo findings reliably inform therapeutic development and personalized treatment strategies.

Taken together, organotypic brain slice cultures represent a biologically sophisticated and experimentally versatile model system. Their continued evolution will depend not only on technological innovation but also on coordinated efforts to enhance methodological transparency, reproducibility, and inter-laboratory validation. Through such efforts, these systems have the potential to become central infrastructures in translational neuro-oncology rather than remaining complementary experimental tools.

## 6 Future perspectives - toward a paradigm shift in translational neuro-oncology

Organotypic brain slice cultures are increasingly moving beyond their original role as intermediate models between *in vitro* and *in vivo* systems. Instead, they offer a way to study glioblastoma within a preserved tissue context that retains key structural and functional features of the brain. Unlike reductionist approaches based on isolated cells, slice cultures maintain elements such as white matter tracts, perivascular niches, glial networks, and components of the local immune environment. This allows tumor cells to interact with their surroundings in a way that more closely reflects *in situ* behavior.

This shift has important implications for how glioblastoma is studied. Traditional models often fail to capture the architectural, mechanical, metabolic, and immunological constraints that shape tumor growth in the brain. By preserving these features, organotypic slices provide a more realistic experimental setting and extend their use beyond descriptive invasion assays toward a platform for studying tumor–microenvironment interactions in a controlled yet physiologically relevant context. To fully realize this potential, further methodological standardization will be necessary. Consistent protocols for tissue preparation, oxygenation, viability assessment, and quantitative analysis are essential if slice cultures are to become reliable tools for translational research. In addition, validation across laboratories and more transparent reporting standards will be important for improving reproducibility and comparability between studies. At the same time, recent technological advances are expanding the scope of what can be achieved with these systems. The combination of ex vivo drug testing with single-cell and spatial transcriptomic approaches makes it possible to track how tumor cells respond to therapy within their native microenvironment. This may help uncover resistance mechanisms that depend on specific anatomical niches, such as perivascular or hypoxic regions, and that are difficult to detect in simpler model systems. Developments in microfluidic platforms and perfusion systems further support this direction by enabling more controlled culture conditions and dynamic monitoring of tumor behavior. Incorporating patient-derived immune cells or immunotherapeutic strategies into these models could also open new avenues for studying immune responses in human brain tissue.

In a clinical context, organotypic human slice cultures may eventually contribute to more individualized treatment strategies. When combined with molecular diagnostics, functional assays performed on patient-derived tissue could provide direct information on treatment sensitivity within clinically relevant timeframes. This would complement genomic profiling by adding a phenotypic layer of evidence, moving closer to a functionally informed approach to precision oncology.

Overall, organotypic brain slice cultures offer a way to study glioblastoma without losing the complexity of the tissue environment in which it arises. By preserving key aspects of brain architecture while remaining experimentally accessible, they provide a valuable bridge between mechanistic research and translational application. With continued technical refinement and integration into multi-modal workflows, they are likely to become an increasingly important component of neuro-oncology research and, potentially, future therapeutic decision-making.

## 7 Conclusion

Organotypic brain tissue slice cultures have developed into a valuable model for studying glioblastoma within a preserved tissue context. By maintaining key structural features such as white matter tracts, perivascular niches, glial networks, and elements of the local immune environment, they capture aspects of tumor biology that are difficult to reproduce in conventional *in vitro* systems. This makes them particularly useful for investigating invasion, tumor–microenvironment interactions, and therapy response under conditions that more closely resemble *in situ* growth.

Rodent-derived slices provide experimental flexibility, genetic accessibility, and a controlled setting for mechanistic studies. In contrast, human slice cultures retain patient-specific architecture, cellular heterogeneity, and clinically relevant molecular features. This difference is important: human slices allow tumor behavior and treatment response to be studied within the actual microenvironment in which they arise. Another strength of slice-based systems is their compatibility with a wide range of analytical approaches, including advanced imaging, electrophysiology, radiobiology, and both spatial and single-cell profiling techniques. Combining these methods with ex vivo perturbation experiments makes it possible to follow how tumor cells respond to treatment within defined tissue regions and microenvironmental niches. At the same time, there are clear limitations. Tissue viability is restricted, systemic influences are absent, and physiological conditions change over time in culture. Nevertheless, ongoing improvements in tissue handling, perfusion systems, and culture protocols are gradually extending viability and improving reproducibility. Greater methodological consistency and clearer reporting standards will be important to ensure that results can be compared across studies and translated more reliably.

Taken together, organotypic brain slice cultures provide a way to study glioblastoma without losing the structural and cellular context of the brain. With continued technical refinement and integration into multi-modal research approaches, they are likely to become an increasingly important tool in both experimental neuro-oncology and the development of more individualized treatment strategies.
